# Altered kinematics and muscle activities during half squats in people with ankle sprain history

**DOI:** 10.1371/journal.pone.0326984

**Published:** 2026-08-13

**Authors:** Umi Matsumura, Rena Ogusu, Shimpei Yamamoto, Toshiya Tsurusaki, Yeonghee Lee, Shinya Sunagawa, Hironobu Koseki

**Affiliations:** 1 Department of Rehabilitation, Social Welfare Corporation Chienkai Sanhaitsu, Nagasaki city, Japan; 2 Department of Health Sciences, Nagasaki University Graduate School of Biomedical Sciences, Nagasaki city, Japan; 3 Department of Rehabilitation, Yurino Hospital, Nishisonogi-Gun Togitsu, Nagasaki, Japan; 4 Department of Physical Therapy Faculty of Nursing and Rehabilitation, Chubu Gakuin University, Seki city, Gifu, Japan; 5 Institute of Biomedical Sciences, Nagasaki University, Nagasaki city, Japan; 6 Department of Rehabilitation, Wajinkai Hospital, Nagasaki city, Japan; 7 Koseki orthopedic clinic, Nagasaki city, Japan; Universita Politecnica delle Marche Facolta di Ingegneria, ITALY

## Abstract

Copers are individuals with a history of ankle sprain who return to daily and sports activities without recurrent instability. However, it is unclear whether copers can use movement strategies comparable to those of healthy individuals without a history of ankle sprain. Understanding these movement strategies may be important for designing effective training programs and avoiding unintended biomechanical adaptations. Therefore, we aimed to compare the kinematics and muscular activities of the lower limbs during half squats and gait between copers and healthy controls. Ten copers (3 females) and 10 controls (4 females) were included in this secondary cross-sectional study. All participants were young, physically active adults who engaged in recreational physical activities for more than 1.5 h per week. We compared mean and peak angles of the thigh, shank, and foot calculated from inertial measurement units, as well as muscle activities of the biceps femoris long head, rectus femoris, gastrocnemius, and tibialis anterior between copers and controls during half squats and gait. During the half squat, copers exhibited lower thigh rotational angles and lower activities of biceps femoris long head, rectus femoris and tibialis anterior across both the descending and ascending phases, accompanied by a higher mean foot angle. In contrast, gait-related group differences were limited to the mean shank angle during the stance phase, with no significant differences observed in other limb angles or muscle activities. These findings suggest that movement strategy alterations in copers are task-dependent and more evident during demanding exercises such as the half squat than during level walking. Identifying such movement characteristics may be important for designing efficient training or rehabilitation programs for individuals with a history of ankle sprain.

## Introduction

Lateral ankle sprain is common injury during sports and daily activities, and about 40% of injured individuals develop chronic ankle instability (CAI) [1,2]. CAI is characterized by repeated episodes of giving way, pain, swelling, limited motion and muscle weakness, which can impair daily and sports activities and may increase the risk of osteoarthritis [3,4]. Following the initial ankle sprains, damaged proprioceptors of the sprained ankle affect neuromuscular strategies of the ankle and the proximal joints [3,5]. Altered muscle activities and kinematics have been reported during functional tasks and gait in people with CAI [1,3,5–10].

However, some individuals do not develop instability after initial ankle sprain and continue their activities without symptoms; these individuals are referred to as “copers” [10]. Even though copers continue their daily activities and resume moderate physical activity [10,11], they still demonstrate altered movement strategies during tasks such as jump landing and single-leg stance [12,13], suggesting that compensatory patterns may persist. However, almost half of the individuals with a history of ankle sprain do not seek medical attention and do not receive proper treatment or rehabilitation [11].

Squatting is a fundamental exercise in rehabilitation and strength training [14,15]. We selected a half squat because it minimizes trunk motion in non-athletic populations and reflects daily functional activities [14,15]. We limited our analysis to the sagittal plane to enhance simplicity and feasibility in clinical settings, as multi-plane analysis increases data volume and noise when using wearable sensors [16]. Wearable sensors such as inertial measurement units (IMU) and electromyography (EMG) allow quantitative evaluation outside the laboratory [16–19].

Although copers are able to return to daily and sports activities without perceived instability, it remains unclear whether they use movement strategies comparable to those of individuals without a history of ankle sprain, particularly during functional exercises. Clarifying these strategies is important for optimizing exercise prescription and preventing inefficient or maladaptive movement patterns. To our knowledge, no study has examined lower-limb kinematics and muscle activities in copers during half squats using wearable sensors. Therefore, we aimed to compare lower-limb kinematics and muscle activities between copers and healthy controls during half squats and gait. We hypothesized that copers would demonstrate altered kinematics and muscle activation patterns during the half squat, but not during gait.

## Materials and methods

### Study design

This study was a secondary cross-sectional analysis conducted at Nagasaki University Graduate School of Biomedical Sciences between 2018 and 2022. Participants were not randomly assigned to groups because classification was based on ankle sprain history [19]. Only healthy control participants overlapped with the primary study. Blinding was not applied due to the observational design; however, objective wearable sensor data and predefined analysis procedures were used to minimize potential bias.

### Participants

The participants were ten copers and ten age- and body-shape-matched healthy controls. All participants provided written informed consent after receiving an explanation of the study. We conducted this study per the Helsinki Declaration’s guidelines, and the Ethics Committee of the Graduate School of Medical Facilities, Nagasaki University, Nagasaki, Japan (Approval number 18061429, 20070905, 21090905).

Following the consensus recommendations by Wikstrom and Brown, the copers were operationally defined as 1) a history of at least one significant ankle sprain with inflammatory symptoms that led to a minimum of one day of interrupted physical activity at least 12 months before enrolling in the study; 2) having more than 1.5 h of physical activity per week; 3) no recurrent ankle sprains within the 3 months before the study; and 4) no previous “giving way” and/or “feelings of instability” on the injured ankle. Feelings of instability were screened using the Ankle Instability Instrument (AII): a “yes” answer to the question “have you ever sprained an ankle?” and a “no” answer to the other questions regarding perceived ankle instability during activities of daily living such as walking or climbing stairs [10,20]. This conservative threshold was selected to ensure that only individuals with no self-reported instability were included. The participants in our study did not report receiving structured rehabilitation intervention or ankle support devices. Therefore, any potential effects of rehabilitation on movement strategies were likely minimal in this cross-sectional study. All participants spent their usual physical activity levels (>1.5 h per week), which included recreational sports participation. Exclusion criteria for both groups were: 1) a history of other musculoskeletal diseases or neurological diseases; 2) pain, inflammation, and swelling that interferes with motions; 3) acute sport-related injury to the lower limb that required rest from physical activities 3 months before the study; and 4) history of surgery or rehabilitation in the lower limb [9].

### Procedures

Participants wore shorts and were barefoot. The squat was performed with a shoulder-width stance, at a comfortable speed, with the arms folded in front of the chest. Participants were instructed to maintain an upright trunk to minimize the effects of head and trunk movements, and to bend their knees as far as possible. We did not prescribe a specific knee flexion angle, because our aim was to capture natural movement strategies rather than impose a uniform movement pattern. Standardizing depth may have masked compensatory strategies, and strict depth control is rarely feasible in clinical practice.

Participants walked straight for 5 m in their natural walking form for the gait task at a comfortable speed while looking forward. All participants performed half-squat and gait tasks thrice. The order of the two tasks (half squat and gait) was randomized across participants to avoid any order effects. A 1-min rest between trials and a 5-min rest between tasks were provided to minimize fatigue. Following the trial, the participants performed a maximum voluntary isometric contraction (MVIC) task.

MVIC was performed separately for each muscle. The MVIC trials for each muscle were performed in the specific limb positions used in manual muscle testing [21]. Two MVIC trials were collected for each muscle, and participants were instructed to exert maximal effort and hold the contraction for 3 s while a physical therapist applied resistance and provided verbal encouragement. The mean value of the two trials, calculated from a 0.5 s window during the plateau phase of maximal effort, was used as the reference MVIC value for normalization [7].

### Data acquisition

Although five copers reported bilateral sprain history and five reported sprains on the right side only, the right limb was measured in all participants to ensure consistency in group comparisons. We calculated lower limb rotation angles using IMUs (LP-WSD1101-OA, 5G/ 300dps, LOGICAL PRODUCT, Fukuoka, Japan) sampled at 1000 Hz. We mounted the IMUs on the lateral aspect of the thigh, shank, and foot using double-sided adhesive tape to measure limb rotation in the sagittal plane based on our previous study [19]. The attachment positions were the thigh at halfway between the greater trochanter and lateral femoral condyle, the shank at halfway between the lateral femoral condyle and lateral malleolus, and the middle of the dorsum of the foot [17–19]. Before data collection, all three IMUs were calibrated in a quiet standing posture. The standing posture was used as the reference position, and all rotational angles were expressed relative to this posture.

We measured muscle activation of the biceps femoris long head (BFL), rectus femoris (RF), gastrocnemius (GAS), and tibialis anterior (TA) using surface EMG (LP-IW2PAD; LOGICAL PRODUCT) sampled at 1000 Hz with a 4-channel system (LP-WSD1002-OA; LOGICAL PRODUCT). We focused on the major muscles that represent ankle and knee movements in the sagittal plane, such as ankle dorsiflexion/plantar flexion and knee extension/flexion. We positioned bipolar Ag/AgCl EMG electrodes (VL-00-S/25; METS, 1–7, Tokyo, Japan) according to the Surface EMG for Non-Invasive Assessment of Muscles guidelines [22] with an interelectrode distance of 30 mm, considering contraction errors in the clinical settings [23]. Before attaching the electrodes, we shaved the skin and disinfected it with alcohol, and a physical therapist palpated the muscle valleys to prevent artifacts from muscle crosstalk.

### Data processing

The limb angle in the sagittal plane was calculated by integrating the angular velocity in the sagittal plane measured using the IMUs with time displacement [16]. We applied a Kalman filter to minimize the common integration error in IMUs, through which the signal was low-passed at 6 Hz, using a zero-phase fourth-order Butterworth filter [16–19].

EMG signals were processed as follows. First, the raw signals were band-pass filtered at 20–450 Hz and then full-wave rectified. The rectified signals were subsequently low-pass filtered at 6 Hz using a fourth-order zero-lag Butterworth filter to obtain the linear envelope and normalized to the reference MVIC value [15]. IMU and EMG envelopes were time-synchronized and then down-sampled to 30 Hz (one sample every 1/30 s) to match the time scale used for analysis [24]. EMG envelopes were low-pass filtered at 6 Hz before down-sampling to avoid signal distortion.

For both groups, the mean values of three trials in half squats and gait were used for analysis. Regarding gait, one gait cycle from the third step was extracted from each of the three trials, because the first two steps were usually part of acceleration and do not reflect a consistent walking pattern. The mean value of these three gait cycles was used for analysis, excluding the first and last cycles to avoid acceleration and deceleration effects. Data for one squat and one gait cycle were converted to 100%. For the half-squat, the descending phase was defined when the shank angle reached its peak, and the ascending phase was defined after reaching the peak. The descending phase lasted 60% of the movement duration, followed by the ascending phase. For the gait, we detected the beginning of the stance and swing phases according to the methods of Watanabe et al [18]. In brief, if the sum of the absolute value of acceleration signals of the three axes exceeded 0.15, it was considered the beginning of the stance. If it fell below 0.15, it was considered the beginning of the swing phase. The stance phase lasted 60% of the movement duration, followed by the swing phase.

### Statistical analyses

We calculated the sample size using G*Power software (version 3.1; RRID:SCR_013726), based on a significance level of 0.05, statistical power of 80%, and an effect size of 0.5, according to Koshino et al. [8]. Eight participants per group were expected to detect a group difference during the task. We included 10 participants per group to compensate for the possibility of defective data. All statistical analyses were performed using JMP Pro version 15 (SAS Institute Inc., Cary, NC, USA). We used the Shapiro–Wilk test to confirm the normal data distribution. We used two-tailed independent t-tests to identify group differences in mean and peak angles and muscle activities during half squat and gat. Statistical significance was set at *p* < 0.05. In addition, we calculated Cohen’s d effect size (ES) to evaluate the magnitude of group differences. We interpreted ES as follows: ≥ 0.80, large; 0.50–0.79, moderate; 0.20–0.49, small; and <0.20, trivial [5,6]. Sex was recorded for all participants. Due to the small sample size, sex was not included as a covariate in the analysis. However, the sex distribution was comparable between the copers (7 females) and the control group (6 females), reducing the likelihood that sex differences biased the group comparisons.

## Results

### Participants characteristics

All participants who met the inclusion criteria completed the study procedures, and no participants were excluded from the analyses. Therefore, no attrition occurred and no observations were lost. There were no significant group differences between copers and controls regarding age, sex, height, weight or body mass index (Table 1). All participants were non-athletes, and several engaged in recreational sports including basketball (n = 3), soccer (n = 2), tennis (n = 2), rugby (n = 2), running (n = 2) and volleyball (n = 1). Copers had a previous ankle sprain an average of 4.9 ± 5.0 years ago (range: 1.7–15.0 years), on their right ankle (measured side).

**Table 1:** Characteristics of copers and controls.

	Copers (n = 10)	Controls (n = 10)
Age, years	21.6 ± 1.1	22.5 ± 2.3
Sex, females	3	4
Height, m	1.68 ± 0.1	1.67 ± 0.1
Weight, kg	63.1 ± 7.0	63.0 ± 15.1
Body mass index, kg/m^2^	22.5 ± 2.8	22.3 ± 3.4

### Lower limb kinematics and muscle activities during the half squat

**Fig 1:**
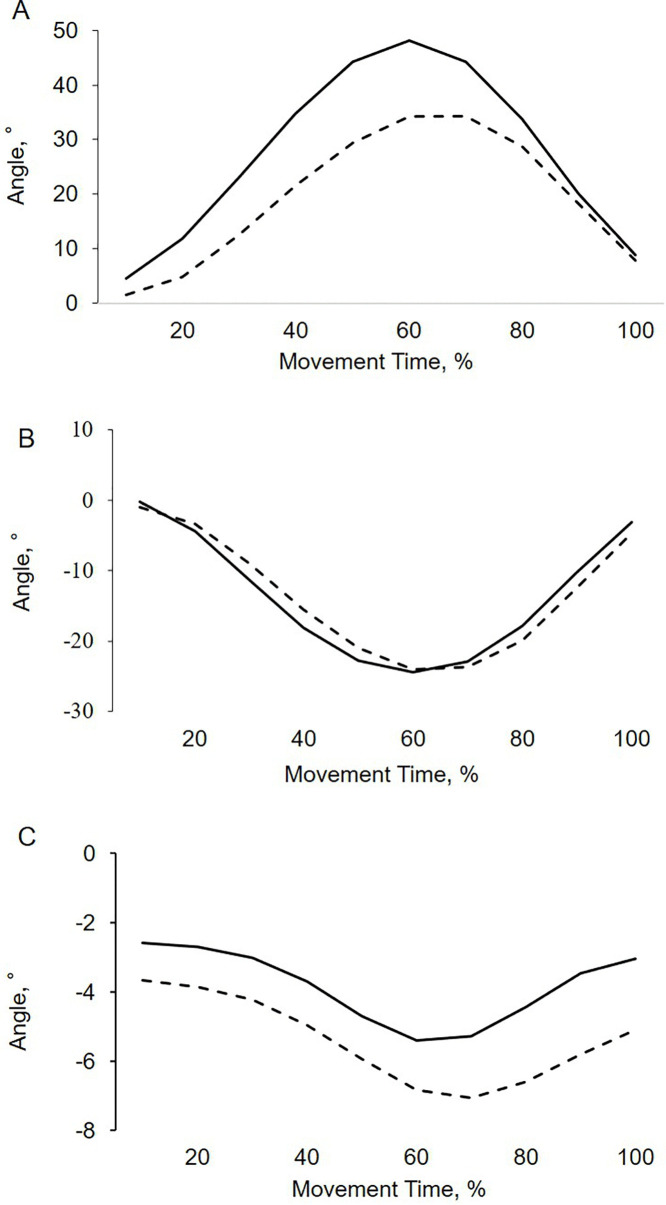
Lower limb angles during a half squat.

**Fig 2:**
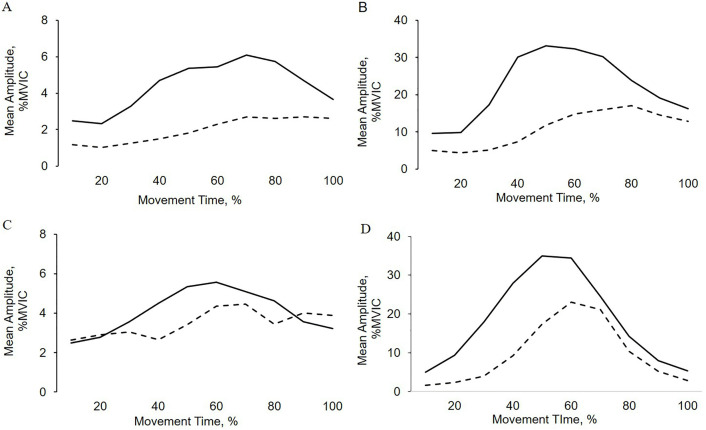
Muscle activities during a half squat.

Fig 1 and Fig 2 illustrate the lower-limb angles and muscle activities during the half squat. Table 2 and 3 summarize the mean and peak values for each phase.

**Table 2:** Mean and peak lower-limb kinematics during the half squat.

		Descending phase				Ascending phase			
		Copers	Controls	p	d	Copers	Controls	p	d
Thigh angle (°)	Mean	17.3 ± 14.6	27.7 ± 19.2	**< 0.05**	0.61	22.3 ± 14.2	26.7 ± 16.0	**< 0.05**	0.29
	Peak	34.3 ± 10.0	48.5 ± 11.1	**< 0.05**	1.35	34.7 ± 10.6	44.3 ± 9.6	**< 0.05**	0.96
Shank angle (°)	Mean	−12.3 ± 9.9	−13.5 ± 13.3	0.33	0.10	−15.1 ± 9.6	−13.5 ± 11.9	0.23	0.16
	Peak	−24.1 ± 6.4	−24.6 ± 13.2	0.83	0.06	−23.9 ± 6.3	−23.0 ± 12.0	0.73	0.09
Foot angle (°)	Mean	−4.9 ± 3.6	−3.7 ± 2.2	**< 0.05**	0.41	−6.1 ± 4.2	−4.1 ± 2.4	**< 0.05**	0.61
	Peak	−6.8 ± 4.1	−5.6 ± 2.2	0.14	0.39	−7.1 ± 4.2	−5.4 ± 2.4	0.06	0.51

Values are presented as mean ± standard deviation. d indicates the absolute value of Cohen’s d effect size. The descending phase corresponded to the first 60% of the squat cycle, followed by the ascending phase.

**Table 3:** Mean and peak muscle activities during the half squat.

		Descending phase				Ascending phase			
		Copers	Controls	p	d	Copers	Controls	p	d
BFL (%MVIC)	Mean	1.5 ± 0.9	3.9 ± 3.2	**< 0.05**	1.04	2.7 ± 1.7	5.0 ± 3.6	**< 0.05**	0.85
	Peak	2.6 ± 1.2	6.4 ± 4.2	**< 0.05**	1.25	3.5 ± 2.0	6.6 ± 4.3	**< 0.05**	0.92
RF (%MVIC)	Mean	8.1 ± 7.3	22.0 ± 18.0	**< 0.05**	1.01	15.1 ± 10.8	22.4 ± 15.6	**< 0.05**	0.54
	Peak	15.2 ± 10.3	39.0 ± 21.4	**< 0.05**	1.42	18.9 ± 12.5	31.5 ± 18.5	**< 0.05**	0.80
GAS (%MVIC)	Mean	3.2 ± 2.7	4.0 ± 4.0	**0.02**	0.26	3.9 ± 2.7	4.1 ± 3.8	0.67	0.28
	Peak	5.5 ± 3.4	6.9 ± 6.1	0.30	0.05	6.1 ± 3.1	6.2 ± 5.4	0.92	0.03
TA (%MVIC)	Mean	9.6 ± 12.0	21.6 ± 19.4	**< 0.05**	0.74	9.9 ± 12.2	13.0 ± 14.2	**< 0.05**	0.24
	Peak	25.5 ± 14.6	43.4 ± 20.4	0.07	1.01	21.8 ± 16.3	25.5 ± 18.3	0.41	0.22

Values are presented as mean ± standard deviation. BFL: Biceps femoris long head; RF: Rectus femoris; GAS: Gastrocnemius; TA: Tibialis anterior; MVIC: maximal voluntary isometric contraction. d indicates the absolute value of Cohen’s d effect size. The descending phase corresponded to the first 60% of the squat cycle, followed by the ascending phase.

Data are presented as mean values of every 10% durations. A) Thigh angle, B) Shank angle, C) Foot angle. Solid line: controls; dotted line: copers. The descending phase corresponded to the first 60% of the squat cycle, followed by the ascending phase.

Data are presented as mean values of every 10% durations. A) Biceps femoris long head, B) Rectus femoris, C) Gastrocnemius, D) Tibialis anterior. Solid line: controls; dotted line: copers; MVIC: maximal voluntary isometric contraction. The descending phase corresponded to the first 60% of the squat cycle, followed by the ascending phase

Across both the descending and ascending phases, copers exhibited significantly lower mean and peak thigh rotational angles compared with healthy controls, whereas shank rotational angles did not differ between groups. In contrast, the mean foot rotational angle was greater in copers during both phases of the half squat.

Regarding muscle activities, copers showed lower mean and peak activities of the BFL and RF across both phases. During the descending phase, additional reductions in TA and GAS activities were observed in copers. Effect sizes ranged from small to large (Tables 2 and 3).

### Lower limb kinematics and muscle activities during the gait

**Fig 3:**
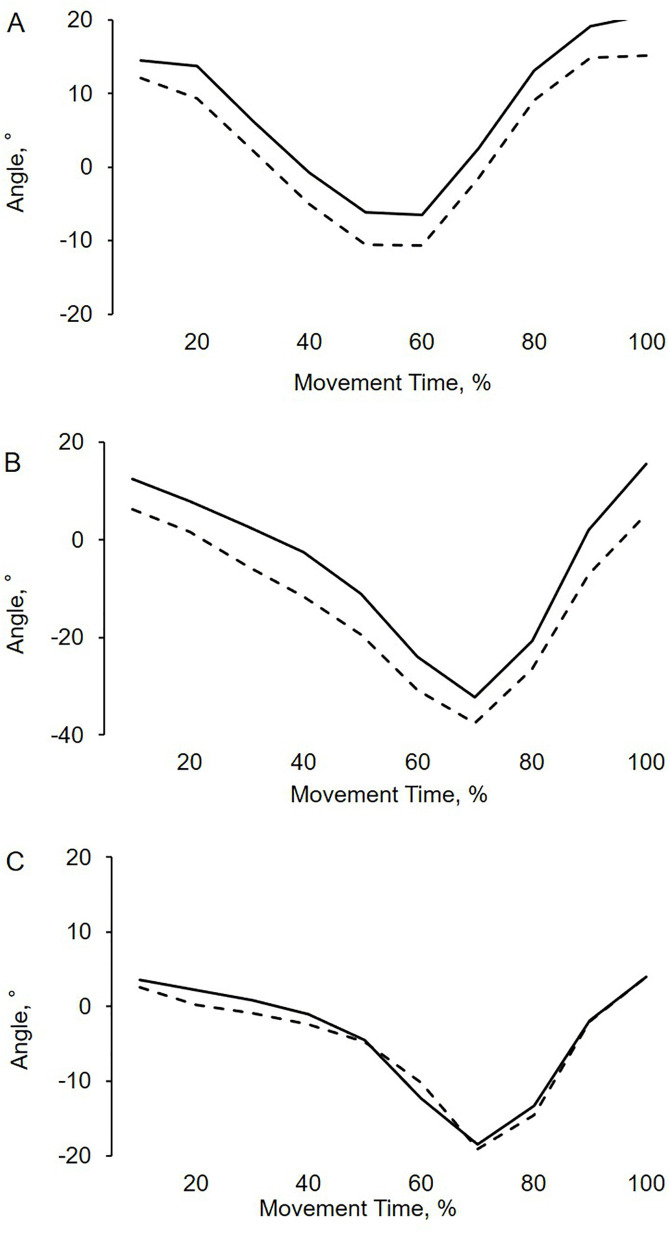
Lower limb angles during gait.

**Fig 4:**
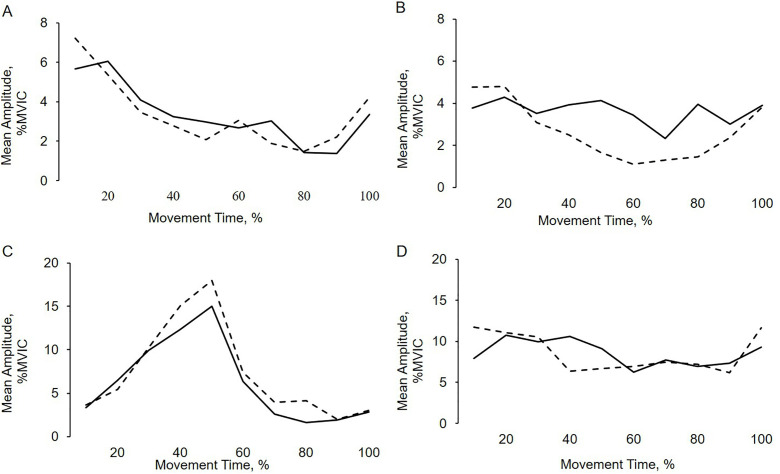
Muscle activities during gait.

Figs 3 and 4 present the lower-limb rotational angles and muscle activities during gait, and Tables 4 and 5 summarize the mean and peak values for each phase.

**Table 4:** Mean and peak lower-limb kinematics during the gait.

		Stance phase				Swing phase			
		Copers	Controls	p	d	Copers	Controls	p	d
Thigh angle (°)	Mean	−0.45 ± 13.8	3.5 ± 10.1	0.09	0.33	9.4 ± 12.2	13.9 ± 11.6	0.11	0.37
	Peak	−12.5 ± 8.7	−7.6 ± 5.4	0.13	0.67	−2.4 ± 11.7	2.5 ± 9.1	0.37	0.46
Shank angle (°)	Mean	−9.9 ± 20.2	−2.4 ± 13.1	**0.02**	0.44	−16.5 ± 24.3	−8.9 ± 23	0.16	0.32
	Peak	−30.8 ± 16.9	−24.0 ± 7.7	0.29	0.52	−41.1 ± 11.3	−34.3 ± 7.5	0.16	0.71
Foot angle (°)	Mean	−2.6 ± 8.8	−1.9 ± 8.1	0.66	0.09	−7.9 ± 13.2	−7.4 ± 13.2	0.87	0.04
	Peak	−9.4 ± 8.4	−12.4 ± 9.8	0.52	0.32	−18.3 ± 12.7	−21.1 ± 8.7	0.61	0.25

Values are presented as mean ± standard deviation. d indicates the absolute value of Cohen’s d effect size. Stance phase were until 60% of the gait cycle, followed by swing phase.

**Table 5:** Mean and peak muscle activities during the gait.

		Stance phase				Swing phase			
		Copers	Controls	p	d	Copers	Controls	p	d
BFL (%MVIC)	Mean	4.0 ± 3.7	4.1 ± 3.9	0.87	0.03	2.5 ± 2.2	2.3 ± 2.5	0.76	0.07
	Peak	8.7 ± 4.6	7.6 ± 4.8	0.65	0.22	5.2 ± 2.4	4.9 ± 3.8	0.87	0.08
RF (%MVIC)	Mean	3.0 ± 2.2	3.8 ± 3.7	0.15	0.28	2.2 ± 2.4	3.3 ± 3.0	0.10	0.38
	Peak	5.4 ± 2.0	5.7 ± 4.5	0.89	0.07	3.9 ± 3.2	5.5 ± 3.9	0.38	0.44
GAS (%MVIC)	Mean	10.0 ± 9.2	8.9 ± 9.6	0.56	0.11	3.3 ± 4.0	2.3 ± 1.6	0.15	0.33
	Peak	21.0 ± 11.1	19.2 ± 15.1	0.78	0.14	7.0 ± 6.5	3.9 ± 2.3	0.20	0.63
TA (%MVIC)	Mean	8.9 ± 10.3	9.1 ± 12.6	0.93	0.02	8.1 ± 4.9	7.8 ± 5.6	0.81	0.06
	Peak	18.3 ± 15.6	14.5 ± 16.8	0.63	0.24	13.4 ± 6.5	10.4 ± 6.8	0.37	0.45

Values are presented as mean ± standard deviation. BFL: Biceps femoris long head; RF: Rectus femoris; GAS: Gastrocnemius; TA: Tibialis anterior; MVIC: maximal voluntary isometric contraction. d indicates the absolute value of Cohen’s d effect size. Stance phase were until 60% of the gait cycle, followed by swing phase.

Data are presented as mean values of every 10% durations. A) Thigh angle, B) Shank angle, C) Foot angle. Solid line: controls; dotted line: copers. The stance phase corresponded to the first 60% of the gait cycle, followed by the swing phase.

Data are presented as mean values of every 10% durations. A) Biceps femoris long head, B) Rectus femoris, C) Gastrocnemius, D) Tibialis anterior. Solid line: controls; dotted line: copers; MVIC: maximal voluntary isometric contraction. The stance phase corresponded to the first 60% of the gait cycle, followed by the swing phase.

During the stance phase, copers demonstrated a significantly lower mean shank rotational angle compared with healthy controls. No significant group differences were observed for thigh or foot rotational angles during stance phase, nor for any rotational angles during the swing phase.

Regarding muscle activities, no significant group differences were observed in the mean or peak values of all four muscles across either the stance or swing phases (p ≥ 0.10). Visual inspection of the waveforms (Fig 3 and Fig 4) suggested that the timings of peak limb angles and peak muscle activations were largely consistent between groups, although the timing of these peaks were not quantitatively analyzed in the present study.

## Discussion

We examined lower limb rotational angles and muscle activities during half squats and gait between copers and healthy controls using IMUs and EMG. The principal finding was that, during the half squat, copers exhibited lower thigh rotational angle and lower EMG amplitudes across both the descending and ascending phases, accompanied by a higher mean foot rotational angle, which supported our hypothesis. In contrast, gait-related group differences were limited, with only a small difference observed in the mean shank rotational angle during stance. These results suggest that altered movement strategies in copers are task-dependent and more evident during the half squat than during level walking.

Across both phases of the half squat, copers exhibited reduced thigh rotational angles without corresponding differences in shank rotation (Table 2), indicating less knee flexion compared with healthy controls. In contrast, copers showed greater foot rotation (Table 2), suggesting a shift toward a more distal, foot-dependent movement strategy during the half squat. This pattern differs from previous findings in individuals with CAI, who often demonstrate increased hip and knee flexion during demanding tasks such as jump landing or cutting maneuvers [8,13,25,26]. Together, these findings suggest that copers may regulate proximal joint motion differently from individuals with persistent instability (individuals with CAI), potentially reflecting a cautious but effective adaptation during functional exercise.

Regarding muscle activity, copers demonstrated lower activation of the BFL and RF across both phases of the half squat, with additional reductions in TA and GAS activities during the descending phase (Table 3). Reduced muscle activation during functional tasks has also been reported in individuals with CAI [1,6,7], suggesting that altered neuromuscular strategies may persist after ankle sprain. However, lower EMG amplitudes should not be interpreted as muscle weakness, as strength was not directly assessed in this study. Rather, these findings may reflect task-specific motor control adaptations associated with reduced thigh motion during the half squat. Notably, no group differences in muscle activity were observed during gait, indicating that altered activation patterns in copers may be more evident during demanding exercises than during level walking.

The limited gait-related group differences observed in this study suggest that level walking at a comfortable speed may not be sufficiently challenging to reveal altered neuromuscular control in copers. In addition, the short walking distance and relatively small, homogeneous sample (young and physically active adults) may have reduced sensitivity to detect subtle between-group differences. Furthermore, gait analysis was based on a limited number of gait cycles obtained from a short walkway, which may have reduced the reliability of gait estimates. Other limitations include the absence of a CAI group, the lack of self-reported functional outcome measures such as the Cumberland Ankle Instability Tool or Foot and Ankle Ability Measure, and the lack of control for sex differences and limb dominance. Despite these limitations, the present findings suggest that identifying movement strategies specific to demanding tasks, such as the half squat, may be important when designing more efficient training or rehabilitation programs for individuals with a history of ankle sprain.

## Conclusions

Copers exhibited a reduced thigh angle, greater foot angle and less muscle activities compared with those exhibited by controls during half squats. Only mean shank angle difference during stance phase was observed during gait. Our findings suggest that identifying such restrictions could be important in designing more efficient training programs. Approximately 50% of copers return to their previous physical activity without appropriate treatment interventions. However, our study suggests the importance of assessing lower limb movements and muscle activities for copers to obtain efficient training effects, even if they do not experience instability and inconvenience in their daily lives.
